# Postoperative Timing of Chemoprophylaxis and Its Impact on Thromboembolism and Bleeding Following Major Abdominal Surgery: A Multicenter Cohort Study

**DOI:** 10.1007/s00268-023-06899-5

**Published:** 2023-02-18

**Authors:** PROTECTinG investigators, VERITAS Collaborative

**Affiliations:** Division of Surgery, Anaesthesia and Procedural Medicine, Austin Hospital, 145 Studley Road, Heidelberg, VIC 3084 Australia

## Abstract

**Background:**

Major abdominal surgery is associated with bleeding and venous thromboembolism (VTE) risks. Chemoprophylaxis prevents VTE but increases bleeding risk. When compared with pre- and intra-operative chemoprophylaxis, recent evidence suggests that starting chemoprophylaxis postoperatively lowers the risk of bleeding without compromising VTE protection. This study investigates whether an optimal window exists in the postoperative period for initiating chemoprophylaxis in patients undergoing major abdominal surgery.

**Methods:**

Analysis of pooled data from four multicenter PROTECTinG studies, which investigated the timing of perioperative chemoprophylaxis on bleeding and VTE outcomes following major abdominal surgery. Patients that commenced chemoprophylaxis postoperatively were separated into quartiles based on timing of administration within the first 24 h post-surgery.

**Results:**

Overall, 4729 (Abdominal visceral resection *N* = 668, cholecystectomies *N* = 573, major ventral hernia repair *N* = 1701, antireflux surgery *N* = 1787) consecutive patients had chemoprophylaxis commenced within 24 h following elective surgery. Baseline characteristics were comparable between quartiles. Across quartiles and within each procedural type, the timing of starting chemoprophylaxis was not associated with bleeding (2.6, 1.7, 2.7 and 3.2%, *p* = 0.130) or clinical VTE (0.8, 0.2, 0.8 and 0.5%, *p* = 0.131), and did not predict their occurrences on multivariate analysis.

**Conclusion:**

Chemoprophylaxis can be safely started at any time within 24 h post-skin closure in major abdominal surgery, without affecting bleeding or VTE risks. This finding encourages the standardization of chemoprophylaxis timing in the postoperative period to pre-defined times during the day to improve workflow efficiency and chemoprophylaxis compliance.

**Supplementary Information:**

The online version contains supplementary material available at 10.1007/s00268-023-06899-5.

## Introduction

Venous thromboembolism (VTE) is an important preventable postoperative complication associated with significant morbidity, mortality and economic burden [1]. Major abdominal surgery incurs a high risk of VTE [2]. Consequently, it is standard practice to administer chemical and mechanical thromboprophylaxis in the perioperative setting [3]. Although chemoprophylaxis decreases VTE risk, it also increases bleeding risk [4]. Thus, when prescribing chemoprophylaxis, the risk and consequences of VTE need to be balanced against that of bleeding. Guidelines exist to facilitate chemoprophylaxis prescription but they do not specify the optimal timing of administration [5].

The PROTECTinG (Perioperative Timing of Elective Chemical Thromboprophylaxis in General Surgery) research group within the VERITAS (Victorian-collaborative for Education, Research, Innovation, Training and Audit by Surgical trainees) collaborative was established to address this gap in perioperative guidelines. Our studies have shown that postoperative chemoprophylaxis is favored over early (preoperative and intraoperative) administration in the settings of elective cholecystectomy [6], major abdominal resection [7], ventral hernia repair [8], and anti-reflux surgery. These findings were subsequently validated in patients with high baseline VTE risk [9], and by meta-analysis of published literature, which focused on patients undergoing major abdominal surgery [10]. Together, these studies have conclusively demonstrated that following major abdominal surgery, postoperative chemoprophylaxis results in a lower risk of bleeding without compromising VTE protection when compared with early administration. Additionally, other studies have demonstrated that therapeutic anticoagulation (using oral agents for patients with atrial fibrillation) can be safely restarted 24 h after skin-closure without an increased bleeding risk [11, 12]. Unfortunately, these studies do not report on VTE endpoints. Therefore, what remains unknown is the optimal timing of starting chemoprophylaxis in the postoperative period following major abdominal surgery.

The relative paucity of evidence directing timing of chemoprophylaxis has contributed to considerable variation in practice between hospitals and surgeons [3]. Furthermore, as identified by the Australian Commission on Safety and Quality in Health Care, despite internationally recognized guidelines recommending VTE risk assessment and provision of thromboprophylaxis, not all patients are receiving this standard-of-care [13]. As highlighted by multiple studies, usage rates are consistently suboptimal [14, 15], with one large study reporting only 62% of at-risk surgical patients receiving appropriate chemoprophylaxis [2]. There are many reasons for poor compliance, however, this is often due to a failure to prescribe or administer chemoprophylaxis during busy clinical schedules. In this context, knowledge of an ideal window for initiating chemoprophylaxis in the postoperative period, if one exists, taking into account bleeding and VTE risks, will greatly facilitate guideline development and standardization of thromboprophylactic practices. Such a system-wide approach will help ensure that all patients receive appropriate chemoprophylaxis in the postoperative period.

Accordingly, we undertook a multicenter study to determine whether an optimal window exists in the postoperative period for commencing chemoprophylaxis in patients undergoing major abdominal surgery.

## Methods

### Study design

A retrospective analysis of pooled data from 4 multicenter studies was conducted by PROTECTinG investigators, which examined the impact of perioperative timing of chemoprophylaxis following elective cholecystectomies [6], abdominal visceral resections [7], major ventral hernia repairs [8], and anti-reflux surgery. Details of these studies are summarized in Table S1-2. Patients were identified from prospectively maintained databases or from each hospital’s administrative database using the Australian Classification of Health Interventions procedural codes [3]. All patients had chemoprophylaxis commenced within 24 h postoperatively. Patients were excluded if under 18 years-of-age, who underwent an emergency procedure, and those who had chemoprophylaxis initiated before skin closure. This study was approved by the Human Research Ethics Committee across all centers.

### Venous thromboembolism prophylaxis

Thromboprophylaxis included both mechanical and chemical methods. Mechanical thromboprophylaxis comprised sequential compression devices (SCD) and thromboembolic deterrent stockings (TEDS). Chemoprophylaxis included subcutaneous injection of either enoxaparin (daily), heparin (twice daily), or dalteparin (daily), at doses adjusted to each patients’ creatinine clearance and weight. Postoperative chemoprophylaxis refers to administration of any of the above anticoagulants at prophylactic doses after skin closure. The type and timing of chemoprophylaxis across all centers for all operations were at the discretion of the treating team. Additionally, chemoprophylaxis was not administered within 12 h of regional anesthesia.

### Data collection and quality assurance

Data was pooled from each PROTECTinG study onto a universal electronic proforma. This included patient demographics, comorbidities, perioperative parameters, operative details, postoperative timing of chemoprophylaxis, postoperative bleeding, and VTE events. As described in each PROTECTinG study, an array of quality assurance measures was undertaken to maximize data accuracy and minimize inter-observer discrepancies [6–8]. Consequently, a random audit of 10% of data fields for each study demonstrated a mean accuracy rate of 98.1% (range 97.7–98.4%), as outlined in Table S1.

### Study endpoints and definitions

All study endpoints were stipulated and defined a priori. Our primary endpoint was postoperative bleeding risk. Secondary endpoints included rates of major bleeding, minor bleeding, reintervention for bleeding, hemoglobin changes post-surgery and 30-day clinical VTE rate. We used Caprini score to evaluate patients’ risk of VTE (≤2: low, 3–4: moderate, ≥5: high risk). We defined clinical VTE as radiologically proven (CTPA, V/Q scintigraphy, venous ultrasound) symptomatic disease occurring within 30 days post-surgery. The criteria for major bleeding included either the need for blood transfusion, reintervention (surgical, endoscopic or radiological), or a >20 g/L fall in hemoglobin from baseline [6–8]. Minor bleeding refers to any bleeding event failing to meet major bleeding criteria. As guided by local hospital policies, oral antiplatelets and anticoagulants (excluding aspirin) were withheld 3 to 7 days prior to surgery. For those patients who required ongoing therapeutic anticoagulation, bridging enoxaparin was used up to 24 h pre-surgery. There was a 4–6-week follow-up period for all patients following discharge.

### Statistical analysis

To determine whether time to starting chemoprophylaxis affected bleeding and VTE risk, we analyzed time as a categorical and continuous variable. For the former, we separated patients into 6 hourly quartiles post-surgery. For the latter, we analyzed time as a continuum in a multivariate analysis. Continuous variables were assessed using the Student’s t-test for 2 variables and analysis of variance (ANOVA) for >2 variables. Categorical variables were assessed using the Fisher’s exact test for 2 variables and chi square for >2 variables. To determine independent predictors of post-operative bleeding and VTE, and account for differences in Australian state-based practices, a hierarchical multivariate logistic regression analysis was undertaken. In this model, covariates were treated as fixed effects, whereas Australian states were treated as a random effect. For 2 variable comparison, a two-tailed *p* < 0.05 and 95% confidence interval (CI) around the odds ratio (OR) that did not cross one was considered statistically significant. For comparison involving  >2 variables, a Bonferroni corrected *p* value was calculated to account for multiple comparisons. Statistical analyses were conducted using Prism v9 (GraphPad Software, San Diego, CA, USA) and R v4.1.0 (R Foundation for Statistical Computing, Vienna, Austria).

### Power calculation

Based on our own data and published studies [3, 4], the overall risk of bleeding following major abdominal surgery in patients who receive postoperative chemoprophylaxis is approximately 3%. We considered a >1.5% absolute risk difference or >50% relative risk difference between study groups as clinically significant. Given that our entire study cohort is divided into quartiles based on the time of starting chemoprophylaxis postoperatively, and therefore we assumed a 1:1:1:1 ratio of sample size within each quartile, the total sample size required was 3200 patients (800 patients per quartile) to detect a 1.5% absolute difference (50% relative difference) in bleeding events between any two quartiles with 80% statistical power (alpha < 0.05).

## Results

### Baseline patient characteristics

In total, we analyzed 4729 consecutive patients who underwent major abdominal surgery and had chemoprophylaxis initiated within the first 24 h post-surgery. These patients were separated into quartiles based on their timing of chemoprophylaxis administration. The median (range) time for each quartile are as follows; Quartile 1: 4.3 (0.0–6.0) hours, quartile 2: 7.5 (6.3–8.6) hours, quartile 3: 14.0 (12.0–15.7) hours, and quartile 4: 21.2 (18.0–24.0) hours. Within all four major abdominal surgery cohorts, anti-reflux surgery (*N* = 1787), ventral hernia repair (*N* = 1701), major visceral resection (*N* = 668) and elective cholecystectomy (*N* = 573), quartiles were largely comparable in demographic, operative and perioperative characteristics (Tables S3-6). Minor but statistically significant differences between quartiles were found with respect to chemoprophylaxis type for the anti-reflux surgery population (Table S3), hernia type and location for the ventral hernia repair population (Table S4), and surgeon seniority for the major visceral resection population (Table S5). Notably, Caprini score, antiplatelet, and anticoagulant use as well as duration of surgery were similar between quartiles for all four major abdominal surgery cohorts.

### Postoperative chemoprophylaxis timing was not associated with bleeding

There were no significant differences in bleeding rates between quartiles for the overall cohort of patients (2.6%, 1.7%, 2.7% and 3.2%, *p* = 0.13). Sub-group analysis for anti-reflux surgery (0.6%, 0.4%, 1.3%, 1.2%, *p* = 0.373), ventral hernia repair (3.3%, 2.9%, 3.6%, 3.8%, *p* = 0.905), major visceral resection (8.6%, 3.5%, 7.9%, 6.2%, *p* = 0.243) and cholecystectomy (0.2%, 0.2%, 0.0%, 3.0%, *p* = 0.064) also did not show significant differences in bleeding rates between quartiles (Table 1). Furthermore, there was no significant difference in major bleeding, minor bleeding, reintervention for bleeding or hemoglobin changes between quartiles in any of these four major abdominal surgery cohorts. Patients with postoperative bleeding had a significantly longer length of stay (mean (SD), 13.3 (18.9) versus 4.2 (9.8) days, *p* < 0.001) compared to non-bleeders. The mean (SD) time to commence chemoprophylaxis post-skin closure was 814.9 (777.8) minutes for bleeders and 689.0 (503.4) minutes for non-bleeders (*p* = 0.058). Overall and within each procedural type, an optimal time for commencing postoperative chemoprophylaxis in which bleeding was significantly lower was not observed.

**Table 1:** Bleeding outcomes by surgery type

Surgery and outcomes	Time from skin closure to postoperative chemoprophylaxis				*p* value
	Quartile 1	Quartile 2	Quartile 3	Quartile 4	
Anti-reflux surgery	*N* = 530	*N* = 465	*N* = 387	*N* = 405	
All bleeding, *n* (%)	3 (0.6)	2 (0.4)	5 (1.3)	5 (1.2)	0.373
Major bleeding, *n* (%)	2 (0.4)	2 (0.4)	2 (0.5)	3 (0.7)	0.879
Minor bleeding, *n* (%)	1 (0.2)	0 (0.0)	3 (0.8)	2 (0.5)	0.217
Reintervention for bleeding, *n* (%)	2 (0.4)	1 (0.2)	1 (0.3)	1 (0.2)	0.965
Hemoglobin drop, g/L, mean (SD)	−35.7 (33.0)	−13.5 (0.7)	−11.2 (12.1)	−30.8 (23.6)	0.359
Ventral hernia repair	*N* = 389	*N* = 384	*N* = 449	*N* = 479	
All bleeding, *n* (%)	13 (3.3)	11 (2.9)	16 (3.6)	18 (3.8)	0.905
Major bleeding, *n* (%)	6 (1.5)	7 (1.8)	3 (0.7)	9 (1.9)	0.409
Minor bleeding, *n* (%)	7 (1.8)	4 (1.0)	13 (2.9)	9 (1.9)	0.280
Reintervention for bleeding, *n* (%)	5 (1.3)	7 (1.8)	1 (0.2)	6 (1.3)	0.160
Hemoglobin drop, g/L, mean (SD)	−19.0 (20.1)	−12.2 (20.2)	−4.6 (8.7)	−17.5 (17.2)	0.302
Major visceral resection	*N* = 163	*N* = 171	*N* = 140	*N* = 194	
All bleeding, *n* (%)	14 (8.6)	6 (3.5)	11 (7.9)	12 (6.2)	0.243
Major bleeding, *n* (%)	11 (6.7)	5 (2.9)	10 (7.1)	11 (5.7)	0.336
Minor bleeding, *n* (%)	3 (1.8)	1 (0.6)	1 (0.7)	1 (0.5)	0.535
Reintervention for bleeding, *n* (%)	4 (2.5)	2 (1.2)	6 (4.3)	3 (1.5)	0.259
Hemoglobin drop, g/L, mean (SD)	−23.8 (15.6)	−25.7 (15.6)	−25.5 (29.4)	−26.9 (18.0)	0.986
Cholecystectomy	*N* = 111	*N* = 153	*N* = 210	*N* = 99	
All bleeding, *n* (%)	1 (0.2)	1 (0.2)	0 (0.0)	3 (3.0)	0.064
Major bleeding, *n* (%)	1 (0.2)	1 (0.2)	0 (0.0)	2 (2.0)	0.257
Minor bleeding, *n* (%)	0 (0.0)	0 (0.0)	0 (0.0)	1 (1.0)	0.187
Reintervention for bleeding, *n* (%)	1 (0.2)	1 (0.2)	0 (0.0)	0 (0.0)	0.482
Hemoglobin drop, g/L, mean (SD)	−15.0 (0.0)	−31.0 (0.0)	−	−15.0 (0.0)	−
Overall cohort	*N* = 1193	*N* = 1173	*N* = 1186	*N* = 1177	
All bleeding, *n* (%)	31 (2.6)	20 (1.7)	32 (2.7)	38 (3.2)	0.130
Major bleeding, *n* (%)	22 (1.8)	15 (1.3)	15 (1.3)	25 (2.1)	0.259
Minor bleeding, *n* (%)	9 (0.8)	5 (0.4)	17 (1.4)	13 (1.1)	0.064
Reintervention for bleeding, *n* (%)	12 (1.0)	11 (0.9)	8 (0.7)	10 (0.8)	0.837
Hemoglobin drop, g/L, mean (SD)	−22.8 (18.8)	−19.5 (16.5)	−11.2 (31.3)	−24.2 (19.0)	0.156

SD: standard deviation, −: Not applicable. Drop in hemoglobin are absolute numbers

### Postoperative chemoprophylaxis timing was not associated with thromboembolism

There was no significant difference in VTE rates between quartiles for postoperative timing of chemoprophylaxis. Clinical VTE occurred in 10 (0.8%), 2 (0.2%), 9 (0.8%) and 6 (0.5%) patients for each quartile (*p* = 0.131). There was similarly no significant variability amongst quartiles when VTE outcomes were separated into DVT and PE (Table 2). Additionally, on subgroup analysis, no association was found between each quartile and VTE risk for anti-reflux surgery (1.3%, 0.2%, 0.8% and 1.0%, *p* = 0.286), ventral hernia repair (0.5%, 0.3%, 0.4% and 0.0%, *p* = 0.490) and major visceral resection (0.6%, 0.0%, 2.1% and 1.0%, *p* = 0.243). A *p*-value was not derived for the cholecystectomy cohort as a VTE event (DVT) occurred in only 1 of the 573 patients. The occurrence of VTE was associated with an extended hospital stay (mean (SD), 12.4 (14.7) versus 4.4 (10.2) days, *p* < 0.001). The mean (SD) time to commence chemoprophylaxis post-skin closure was 713.0 (630.4) minutes for patients who developed VTE and 692.1 (511.8) minutes for those free from VTE (*p* = 0.833). Overall and within each procedural type, an optimal time for commencing postoperative chemoprophylaxis in which VTE rates were significantly lower was not observed.

**Table 2:** Venous thromboembolism by surgery type

Surgery and outcomes	Time from skin closure to postoperative chemoprophylaxis				*P *value
	Quartile 1	Quartile 2	Quartile 3	Quartile 4	
Anti-reflux surgery	530	465	387	405	
Venous thromboembolism, *n* (%)	7 (1.3)	1 (0.2)	3 (0.8)	4 (1.0)	0.286
Deep vein thrombosis, *n* (%)	4 (0.8)	0 (0.0)	2 (0.5)	1 (0.2)	0.262
Pulmonary embolism, *n* (%)	4 (0.8)	1 (0.2)	1 (0.3)	4 (1.0)	0.345
Ventral hernia repair	389	384	449	479	
Venous thromboembolism, *n* (%)	2 (0.5)	1 (0.3)	2 (0.4)	0 (0.0)	0.490
Deep vein thrombosis, *n* (%)	1 (0.3)	1 (0.3)	2 (0.4)	0 (0.0)	0.574
Pulmonary embolism, *n* (%)	1 (0.3)	1 (0.3)	1 (0.2)	0 (0.0)	0.753
Major visceral resection	163	171	140	194	
Venous thromboembolism, *n* (%)	1 (0.6)	0 (0.0)	3 (2.1)	2 (1.0)	0.243
Deep vein thrombosis, *n* (%)	0 (0.0)	0 (0.0)	1 (0.7)	2 (1.0)	0.362
Pulmonary embolism, *n* (%)	1 (0.6)	0 (0.0)	3 (2.1)	1 (0.5)	0.162
Cholecystectomy	111	153	210	99	
Venous thromboembolism, *n* (%)	0 (0.0)	0 (0.0)	1 (0.5)	0 (0.0)	−
Deep vein thrombosis, *n* (%)	0 (0.0)	0 (0.0)	1 (0.5)	0 (0.0)	−
Pulmonary embolism, *n* (%)	0 (0.0)	0 (0.0)	0 (0.0)	0 (0.0)	−
Overall cohort	1193	1173	1186	1177	
Venous thromboembolism, *n* (%)	10 (0.8)	2 (0.2)	9 (0.8)	6 (0.5)	0.131
Deep vein thrombosis, *n* (%)	5 (0.4)	1 (0.1)	6 (0.5)	3 (0.3)	0.276
Pulmonary embolism, *n* (%)	6 (0.5)	2 (0.2)	5 (0.4)	5 (0.4)	0.583

−: Not applicable

### Predictor of postoperative bleeding

Univariate analysis identified the following factors to be significantly associated with postoperative bleeding: older age, male gender, higher Caprini score, anesthesia type, surgical approach, higher ASA score, longer surgical duration, pre-existing antiplatelet or anticoagulant use, and repeated chemoprophylaxis dosing (Table 3). Of these, multivariate analysis identified older age (OR 1.02, 95% CI 1.01–1.04, *p* = 0.002), longer surgical duration (OR 1.89, 95% CI 1.29–2.77, *p* = 0.001), and pre-existing anti-platelet use (OR 1.69, 95%CI 1.09–2.62, *p* = 0.020) to be independent predictors of postoperative bleeding following major abdominal surgery. Importantly, chemoprophylaxis timing expressed as a categorical or continuous variable was not associated with, or predictive of, postoperative bleeding on univariate or multivariate analysis, respectively.

**Table 3:** Predictors of postoperative bleeding

Characteristics	Bleeding *N* = 121	No Bleeding *N* = 4608	Univariate	Multivariate		
			*p* value	OR	95% CI	*p* value
Demographics						
Age, mean (SD)	65.5 (14.0)	59.4 (14.7)	**<0.001**	1.02	1.01–1.04	0.002
Gender, male, *n* (%)	67 (55.4)	1934 (42.0)	**0.004**	−	−	−
Body mass index, kg/m^2^, mean (SD)	30.0 (7.5)	30.5 (6.4)	0.377	−	−	−
Caprini score, median (IQR)	6 (4–7)	5 (4–6)	**<0.001**	−	−	−
Operative details						
Anesthesia type, *n* (%)			**<0.001**	−	−	−
General only	102 (84.3)	4377 (95.0)				
Regional only	1 (0.8)	9 (0.2)				
General and regional	18 (14.9)	222 (4.8)				
Operation type, *n* (%)			**<0.001**			
Major ventral hernia repair	58 (47.9)	1643 (35.7)		5.29	2.92–9.57	<0.001
Major visceral resection	43 (35.5)	625 (13.6)		5.80	3.11–10.8	<0.001
Cholecystectomy	5 (4.1)	568 (12.3)		1.14	0.32–4.05	0.800
Anti-reflux surgery	15 (12.4)	1772 (38.5)		−	−	−
Operative approach, *n* (%)			**<0.001**	−	−	−
Open	73 (60.3)	1669 (36.2)				
Laparoscopic	46 (38.0)	2902 (63.0)				
Open conversion	2 (1.7)	37 (0.8)				
Surgeon seniority, *n* (%)			0.596	−	−	−
Consultant	84 (69.4)	2985 (64.8)				
Fellow	15 (12.4)	771 (16.7)				
Registrar	21 (17.4)	795 (17.3)				
Not documented	1 (0.8)	57 (1.2)				
Perioperative details						
ASA score, median (IQR)	3 (2–3)	2 (2–3)	**<0.001**	−	−	−
Surgical duration, min, mean (SD)	209.3 (159.2)	146.0 (89.1)	**<0.001**	1.89	1.29–2.77	0.001
Length-of-stay, days, mean (SD)	13.3 (18.9)	4.2 (9.8)	**<0.001**	−	−	−
Antiplatelet agent use, yes, *n* (%)	30 (24.8)	585 (12.7)	**<0.001**	1.69	1.09–2.62	0.020
Anticoagulant agent use, yes, *n* (%)	12 (9.9)	197 (4.3)	**0.011**	−	−	−
Mechanical thromboprophylaxis, yes, *n* (%)	117 (96.7)	4469 (97.0)	0.786	−	−	−
Chemoprophylaxis type, LMWH, *n* (%)	102 (84.3)	4009 (87.0)	0.411	−	−	−
Repeated chemoprophylaxis dosing, yes, *n* (%)	106 (87.6)	3249 (70.5)	**<0.001**	−	−	−
Time to first postop chemoprophylaxis, *n* (%)			0.130			
Quartile 1	31 (25.6)	1162 (25.2)		−	−	−
Quartile 2	20 (16.5)	1153 (25.0)		0.68	0.38–1.21	0.200
Quartile 3	32 (26.4)	1154 (25.0)		1.09	0.65–1.82	0.700
Quartile 4	38 (31.4)	1139 (24.7)		1.03	0.63–1.69	0.999
Time to first postop chemoprophylaxis, min, mean (SD)	814.9 (777.8)	689.0 (503.4)	0.058	−	−	−

Bold indicates significance at *p* < 0.05

*ASA* American Society of Anesthesiology, *CI* confidence interval, *IQR* Interquartile range, *LMWH* Low molecular weight heparin, *OR* odds ratio, *SD* Standard deviation

### Predictor of postoperative venous thromboembolism

Factors that were significantly associated with postoperative VTE on univariate analysis included older age, higher Caprini score, higher ASA score, longer surgical duration, and repeated chemoprophylaxis dosing (Table 4). Multivariate analysis demonstrated higher Caprini score (OR 1.20, 95% CI 1.07–1.33, *p* = 0.001) and longer surgical duration (OR 2.20, 95% CI 1.16–4.17, *p* = 0.016) to be independent predictors of clinical VTE following major abdominal surgery. Similarly, chemoprophylaxis timing expressed as a categorical or continuous variable was not associated with, or predictive of, postoperative VTE on univariate or multivariate analysis, respectively.

**Table 4:** Predictors of venous thromboembolism

Characteristics	VTE *N* = 27	No VTE N4702	Univariate	Multivariate		
			*p* value	OR	95% CI	*p* value
*Demographics*						
Age, mean (SD)	65.1 (15.5)	59.5 (14.7)	**0.048**	−	−	−
Gender, male, *n* (%)	14 (51.9)	1987 (42.3)	0.334	−	−	−
Body mass index, kg/m^2^, mean (SD)	31.2 (6.4)	30.5 (6.4)	0.603	−	−	−
Caprini score, median (IQR)	6 (5–8)	5 (4–6)	**<0.001**	1.20	1.07–1.33	0.001
Operative details						
Anesthesia type, *n* (%)			0.067	−	−	−
General only	23 (85.2)	4456 (94.8)				
Regional only	0 (0.0)	10 (0.2)				
General and regional	4 (14.8)	236 (5.0)				
Operation type, *n* (%)			0.080	–	−	−
Major ventral hernia repair	5 (18.5)	1696 (36.1)				
Major visceral resection	6 (22.2)	662 (14.1)				
Cholecystectomy	1 (3.7)	572 (12.2)				
Anti−reflux surgery	15 (55.6)	1772 (37.7)				
Operative approach, *n* (%)			0.893	−	−	−
Open	10 (37.0)	1732 (36.8)				
Laparoscopic	17 (63.0)	2931 (62.3)				
Open conversion	0 (0.0)	39 (0.8)				
*Surgeon seniority, n (%)*			0.423	−	−	−
Consultant	20 (74.1)	3049 (64.8)				
Fellow	3 (11.1)	783 (16.7)				
Registrar	3 (11.1)	813 (17.3)				
Not documented	1 (3.7)	57 (1.2)				
*Perioperative details*						
ASA score, median (IQR)	3 (2–3)	2 (2–3)	**0.045**	−	−	−
Surgical duration, min, mean (SD)	205.0 (129.4)	147.3 (91.7)	**0.001**	2.20	1.16–4.17	0.016
Length-of-stay, days, mean (SD)	12.4 (14.7)	4.4 (10.2)	**<0.001**	−	−	−
Antiplatelet agent use, yes, *n* (%)	2 (7.4)	613 (13.0)	0.568	−	−	−
Anticoagulant agent use, yes, *n* (%)	3 (11.1)	206 (4.4)	0.115	−	−	−
Mechanical thromboprophylaxis, yes, *n (%)*	26 (96.3)	4560 (97.0)	0.565	−	−	−
Chemoprophylaxis type, LMWH, *n* (%)	23 (85.2)	4087 (86.9)	0.773	−	−	−
Repeated chemoprophylaxis dosing, yes, *n* (%)	25 (92.6)	3330 (70.8)	**0.010**	−	−	−
*Time to first postop chemoprophylaxis, n (%)*			0.131			
Quartile 1	10 (37.0)	1183 (25.2)		−	−	−
Quartile 2	2 (7.4)	1171 (24.9)		0.22	0.05–1.03	0.055
Quartile 3	9 (33.3)	1177 (25.0)		1.03	0.41–2.58	0.999
Quartile 4	6 (22.2)	1171 (24.9)		0.60	1.16–4.17	0.300
Time to first postop chemoprophylaxis, min, mean (SD)	713.0 (630.4)	692.1 (511.8)	0.833	−	−	−

Bold indicates significance at *p* < 0.05

*ASA* American Society of Anesthesiology, *CI* confidence interval, *IQR* Interquartile range, *LMWH* Low molecular weight heparin, *OR* odds ratio, *SD* Standard deviation

## Discussion

This is the first study to investigate the optimal postoperative timing of chemoprophylaxis in major abdominal surgery. Our incidence of clinical VTE and bleeding are comparable to published data on anti-reflux surgery [16–19], ventral hernia repair [20–23], major visceral resection [24–26], cholecystectomy [20, 27–32], and elective general surgery [33–35]. Whilst VTE risk necessitates chemoprophylaxis in the perioperative setting, strong evidence now exists demonstrating that chemoprophylaxis confers a clinically significant risk of bleeding [4]. Multiple studies now show that postoperative commencement of chemoprophylaxis is favored over pre- and intra-operative administration because of reduced bleeding risk while preserving VTE protection [24, 26, 36–39]. In this study, our findings indicate that there is *no* optimal time window for initiating chemoprophylaxis in the early postoperative period. As long as it is given within 24 h of skin closure, both VTE and bleeding risks remain acceptably low.

The significance of this finding is that it informs protocol development towards standardization of thromboprophylactic practices. The current variability in practice likely contributes to the recognized suboptimal compliance with administering chemoprophylaxis prescriptions [40, 41]. Furthermore, non-compliance has been cited as a major reason for surgeons prescribing chemoprophylaxis preoperatively (a practice associated with increased risk of bleeding and no additional VTE protection) [42]. We propose that protocolizing chemoprophylaxis on the surgical ward to set times during the day will likely be easier for nursing staff to manage. With this approach, should an operation finish after one set time, chemoprophylaxis will be given at the next designated time point. This method is different to surgeons requesting chemoprophylaxis to start at fixed intervals postoperatively, as the latter will result in multiple inpatients needing chemoprophylaxis administered at multiple, and often at odd hours of the day (or night). Such practices may lead to confusion and neglect, particularly during periods of high clinical demand, thus compromising adequate thromboprophylaxis. In contrast, standardizing postoperative chemoprophylaxis to specific times during the day fosters routine, enables integration into pre-existing workflows, and allows nursing staff to anticipate and plan, which ultimately may improve compliance to thromboprophylaxis, and patient safety.

There are inherent limitations within this retrospective study. Moreover, we did not search for an optimal chemoprophylaxis window beyond 24 h of skin closure. This is because evidence already exist demonstrating the safety of commencing therapeutic anticoagulation at 24 h post-surgery [11], and it is uncommon for clinicians to delay chemoprophylaxis beyond 24 h post-surgery unless there were specific concerns for bleeding. Additionally, the incidence of clinical VTE may be underestimated in this study as patients may present to other health services for treatment. However, all patients were followed-up between 4–6 weeks post-surgery, and as noted earlier, our reported rates of clinical VTE are comparable to contemporary series [33–35]. We are unable to provide 90-day bleeding or clinical VTE rates as patients were not universally followed-up after 6 weeks post-surgery.

## Conclusion

The timing of initiating chemoprophylaxis within 24 h postoperatively is not significantly associated with bleeding and clinical VTE after major abdominal surgery. Given the low risk of bleeding and clinical VTE associated with postoperative chemoprophylaxis, our findings advocate for standardization of chemoprophylaxis timing in the postoperative period to improve compliance with administration.

## Supplementary Information

Below is the link to the electronic supplementary material.

Supplementary file1 (DOCX 54 kb)
