# Supplementary material for: Postoperative Timing of Chemoprophylaxis and Its Impact on Thromboembolism and Bleeding Following Major Abdominal Surgery: A Multicenter Cohort Study
Source: World J Surg. 2023 Feb 18;47(5):1174–83. doi: 10.1007/s00268-023-06899-5 (PMC10070293; doi:10.1007/s00268-023-06899-5)
Supplement: Supplementary file 1 — Supplementary file1 (DOCX 54 kb) [file 268_2023_6899_MOESM1_ESM.docx]

## Supplementary material

- **Appendix S1:** Authorship
- **Appendix S2:** Investigators’ role and their description
- **Table S1.** Summary of PROTECTinG studies
- **Table S2.** Participating Australian sites
- **Table S3.** Baseline characteristics for anti-reflux surgery cohort
- **Table S4.** Baseline characteristics for major ventral hernia surgery cohort
- **Table S5.** Baseline characteristics for major abdominal visceral resection cohort
- **Table S6.** Baseline characteristics for cholecystectomy cohort

**Appendix S1: Authorship (all co-authors are PubMed citable)**

**PROTECTinG Investigators, VERITAS collaborative**

**Coordinating Principal Investigator**: David S. Liu

**Statistical analysis**: David S. Liu, Darren J. Wong

**Database design:** Su Kah Goh, David S. Liu

**Writing group**: Laura Barbis, David S. Liu, Darren J. Wong, David I. Watson, Enoch Wong, Jonathan Fong, Sean Stevens, Su Kah Goh

**Consultant supervisors**: Ahmad Aly, Vijayaragavan Muralidharan (*Austin Health*). George Kalogeropoulos (*Barwon Health).* Chon Hann Liew (*Bendigo Health*). Sean Stevens (*Colac Area Health, Albury-Wodonga Hospitals*), Salena Ward (*Eastern Health*). Sanjeeva Kariyawasam (*Fiona Stanley Hospital*). Paul Cashin (*Monash Health*). Krinal Mori (*Northern Health*). Matthew Read (*St Vincent’s Hospital, Melbourne*). David I. Watson, Tim Bright (*Flinders Medical Centre and Flinders University, South Australian Hospitals*). Jonathan Fong (*Tasmanian Health*). Adam Cichowitz (*Wangaratta Hospital*). Justin Yeung (*Western Health*).

**Hospital leads**: Daniel Cox, Sean Stevens (*Austin Health*). Jonathon Holt (*Albury-Wodonga Health*), Sara Mohammed Jinnaah (*Barwon Health*). Amy Crowe (*Bendigo Health*). Anh N. Vu (*Colac Area Health*). Enoch Wong (*Eastern Health*). Melissa Y. Wee (*Flinders Medical Centre and Flinders University, South Australian Hospitals*). Marwan Idrees (*Fiona Stanley Hospital*). Geraldine Ooi (*Monash Health*). Sharon Lee (*Northern Health*). Lynn Chong (*St Vincent’s Hospital, Melbourne*). Emma Downie (*Wangaratta Hospital*). Brianne Lauritz (*Western Health*).

**Local collaborators**: Anna S. Gill, Hamza Ashraf, Hein Maung, Kat Hall, Lobna Alukaidey, Nicola Fleming, Samantha Wong, Simon Bennet, Tess Howard, Wael Jamel (*Austin Health*). Caitlin Reid, Hugh Elbourne, Jed Hughes (*Albury-Wodonga Health*). Emily Doole, Gabriel Lirios, Manoj Anandan, Shipra Sankpal, Zoe Zhang (*Barwon Health*). Brett Larner, Emily Fitt, Jessica Paynter, Natalie Guiney, Nevin Chen, Patrick Brown, Ra Nasser (*Bendigo Health*). [Joe Ibrahim](mailto:joe.ibrahim.32@gmail.com)(*Colac Area Health*). Amie Hilder, Anshini Jain, Chen Lew, Chi Chung, Elisa Lie, Gamze Aksakal, James C. Sorensen, King Tung Cheung, Michelle Yao, Olivia Miki Lin, Poojani Pathirana, Surabhi Shashishekara, Shantanu Joglekar, Thomas Bedford (*Eastern Health*). Amanda Hii, James P Grantham, Stephanie G Ng, Steven Tran, Zhi Tan (*Flinders Medical Centre and Flinders University, South Australian Hospitals*). Amy Leathersich, Deanna Lee, Thuc Nhi Lu, Wanyang Qian, Vigneshkumar Palanisamy (*Fiona Stanley Hospital*). Ashray Rajagopalan, Jack Menzie, Jasprit Singh, Sean Lim, Sandy Su (*Monash Health*). Kay Tai Choy, Maeve Slevin, Pith S. Beh, Wael Jamel (*Northern Health*). Emma Choong, James Gray (*St Vincent’s Hospital, Melbourne*). Lily Bae, Tiffany Cherry, Roshini Nadaraja (*Tasmanian Health*). James May, Jonathan McCafferty, Jordan Lee, Sally Wilkinson (*Wangaratta Hospital*). Cheuk Shan Choi, Felicia Ching Siew Ho, Jing Qiao, Francesca Sasanelli, Kyle Bennett, Luke Fairweather, Tazvir Zaman, Walter Santucci, Vivek Jayapadman (*Western Health*).

**Appendix S2: Investigators’ role and their description**

**Coordinating Principal Investigator’s role**: study conception and design, data analysis and interpretation, overall supervision, drafting of article, review and final article approval

**Statistician’s role:** study design, data analysis and interpretation, drafting, review and final article approval

**Database designer’s role:** REDCap database design, testing, final production, drafting, review and final article approval

**Writing group’s role**: data analysis and interpretation, drafting of article, drafting, review and final article approval

**Consultant supervisor’s role:** project governance, site supervision, drafting, review and final article approval

**Hospital lead’s role:** site coordination, project governance, data acquisition, drafting, review and final article approval

**Local collaborator’s role**: site coordination, data acquisition, drafting, review and final article approval

**Table S1.** Summary of PROTECTinG studies for pooled analysis

| **Year** | **Surgery**  **type** | **Study**  **Period** | **Number of**  **centers** | **Data**  **Accuracy (%)*** |
| --- | --- | --- | --- | --- |
| 2022 | Antireflux surgery | 01/01/2010 - 31/12/2020 | 36 | 98.4% |
| 2022 | Major ventral hernia repair | 01/01/2014 - 31/12/2019 | 14 | 97.7% |
| 2022 | Abdominal visceral resection | 01/01/2018 -30/06/2019 | 7 | 98.0% |
| 2020 | Cholecystectomy | 01/01/2018 - 30/06/2019 | 7 | 98.0% |

* Data accuracy based on a random audit of 10% of the entire dataset.

**Table S2. Participating Australian sites**

| **Study name** | **Australian States** | **Hospital name** |
| --- | --- | --- |
| PROTECTinG –  Cholecystectomy  Bariatric surgery  Abdominal visceral resection | Victoria | - Austin Health, Austin Hospital, - Austin Health, Heidelberg Repatriation Hospital - Eastern Health, Box Hill Hospital - Eastern Health, Maroondah Hospital - Eastern Health, Angliss Hospital - Northern Health, The Northern Hospital - Northern Health, Broadmeadows Hospital |
| PROTECTinG –  Major ventral hernia repair | Victoria | - Austin Health, Austin Hospital, - Austin Health, Heidelberg Repatriation Hospital - Bendigo Health, Bendigo Hospital - Eastern Health, Box Hill Hospital - Eastern Health, Maroondah Hospital - Eastern Health, Angliss Hospital - Northern Health, The Northern Hospital - Northern Health, Broadmeadows Hospital |
|  | South Australia | - Flinders Medical Centre - Noarlunga Hospital |
|  | Tasmania | - Launceston General Hospital - Mersey Community Hospital - Northwest Regional Hospital - Royal Hobart Hospital |
| PROTECTinG –  Antireflux surgery | Victoria | - Austin Health, Austin Hospital, - Austin Health, Heidelberg Repatriation Hospital - Barwon Health, Geelong Hospital - Bendigo Health, Bendigo Hospital - Colac Area Health, Colac Regional Hospital - Eastern Health, Box Hill Hospital - Eastern Health, Maroondah Hospital - Eastern Health, Angliss Hospital - Monash Health, Monash Hospital - Monash Health, Dandenong Hospital - Northern Health, The Northern Hospital - Northern Health, Broadmeadows Hospital - Northeast Health, Wangaratta Hospital - St Vincents Health, St Vincent’s Hospital, Melbourne - Western Health, Footscray Hospital - Western Health, Sunshine Hospital |
|  | South Australia | - Ashford Private Hospital - Burnside War Memorial Hospital - Calvary North Adelaide Hospital - Flinders Medical Centre - Flinders Private Hospital - Lyell McEwin Hospital - Noarlunga Hospital - North East Community Hospital - Queen Elizabeth Hospital - Royal Adelaide Hospital - Repatriation General Hospital - St Andrews Hospital - Wakefield Hospital - Whyalla Hospital |
|  | Tasmania | - Launceston General Hospital - Mersey Community Hospital - Northwest Regional Hospital - Royal Hobart Hospital |
|  | New South Wales | - Albury-Wodonga Health |
|  | Western Australia | - Fiona Stanley Hospital |

**Table S3.** Baseline characteristics for anti-reflux surgery cohort

| **Characteristics** | **Time from skin closure to postoperative chemoprophylaxis** | | | | ***P***  **value** |
| --- | --- | --- | --- | --- | --- |
|  | **Quartile 1**  **N=530** | **Quartile 2**  **N=465** | **Quartile 3**  **N=387** | **Quartile 4**  **N=405** |  |
| **Demographics** |  |  |  |  |  |
| Age, mean (SD) | 59.8 (14.2) | 60.0 (14.3) | 59.0 (15.4) | 62.2 (14.5) | 0.338 |
| Gender, male, n (%) | 194 (36.6) | 172 (37.0) | 147 (38.0) | 130 (32.1) | 0.306 |
| Body mass index, kg/m^2^, mean (SD) | 30.2 (5.8) | 29.8 (4.9) | 30.2 (5.3) | 30.0 (5.8) | 0.754 |
| Caprini score, median (IQR) | 5 (4-6) | 5 (4-6) | 5 (4-6) | 5 (4-6) | 0.111 |
| **Operative details** |  |  |  |  |  |
| Anesthesia type, n (%) |  |  |  |  | 0.237 |
| General only | 520 (98.1) | 454 (97.6) | 378 (97.7) | 389 (96.0) |  |
| General and regional | 10 (1.9) | 11 (2.4) | 9 (2.3) | 16 (4.0) |  |
| Operative approach, n (%) |  |  |  |  | 0.576 |
| Open | 19 (3.6) | 13 (2.8) | 18 (4.7) | 27 (6.7) |  |
| Laparoscopic | 502 (94.7) | 445 (95.7) | 358 (92.5) | 366 (90.4) |  |
| Open conversion | 9 (1.7) | 7 (1.5) | 11 (2.8) | 12 (3.0) |  |
| Surgeon seniority, n (%) |  |  |  |  | 0.721 |
| Consultant | 417 (78.7) | 359 (77.2) | 318 (82.2) | 324 (80.0) |  |
| Fellow | 93 (17.5) | 92 (19.8) | 59 (15.2) | 71 (17.5) |  |
| Registrar | 14 (2.6) | 8 (1.7) | 6 (1.6) | 5 (1.2) |  |
| Not documented | 6 (1.1) | 6 (1.3) | 4 (1.0) | 5 (1.2) |  |
| Hernia present, n (%) | 477 (90.0) | 415 (89.2) | 341 (88.1) | 362 (89.4) | 0.840 |
| Hernia size, n (%) |  |  |  |  | 0.102 |
| Small-Medium (<5 cm) | 154 (32.3) | 154 (37.1) | 134 (39.3) | 123 (34.0) |  |
| Large (>5 cm) | 149 (31.2) | 147 (35.4) | 103 (30.2) | 129 (35.6) |  |
| Intrathoracic stomach | 67 (14.0) | 50 (12.0) | 45 (13.2) | 52 (14.4) |  |
| Not documented | 107 (22.4) | 64 (15.4) | 59 (17.3) | 58 (16.0) |  |
| Fundoplication, yes, n (%) | 530 (100.0) | 465 (100.0) | 387 (100.0) | 405 (100.0) | 1.000 |
| Fundoplication type, n (%) |  |  |  |  | 0.312 |
| Complete | 90 (17.0) | 64 (13.8) | 64 (16.5) | 87 (21.5) |  |
| Partial | 440 (83.0) | 401 (86.2) | 323 (83.5) | 318 (78.5) |  |
| Crural repair, yes, n (%) | 530 (100.0) | 465 (100.0) | 387 (100.0) | 405 (100.0) | 1.000 |
| Crural repair site, n (%) |  |  |  |  | 0.345 |
| Anterior | 28 (5.3) | 23 (4.9) | 19 (4.9) | 22 (5.4) |  |
| Posterior | 283 (53.4) | 263 (56.6) | 203 (52.5) | 245 (60.5) |  |
| Anterior and posterior | 174 (32.8) | 150 (32.3) | 133 (34.4) | 106 (26.2) |  |
| Not documented | 45 (8.5) | 29 (6.2) | 32 (8.3) | 32 (7.9) |  |
| Revisional surgery, yes, n (%) | 32 (6.0) | 36 (7.7) | 31 (8.0) | 33 (8.1) | 0.560 |
| Short gastric vessel divided, yes, n (%) | 119 (22.5) | 138 (29.7) | 98 (25.3) | 97 (24.0) | 0.488 |
| **Perioperative details** |  |  |  |  |  |
| ASA score, median (IQR) | 2 (2-3) | 2 (2-3) | 2 (2-3) | 2 (2-3) | 0.130 |
| Surgical duration, min, mean (SD) | 161.9 (73.3) | 159.9 (60.2) | 148.3 (77.0) | 162.6 (74.9) | 0.100 |
| Length-of-stay, days, mean (SD) | 3.6 (5.9) | 3.5 (5.7) | 4.2 (7.8) | 4.6 (4.6) | 0.361 |
| Antiplatelet agent, yes, n (%) | 58 (10.9) | 50 (10.8) | 49 (12.7) | 64 (15.8) | 0.088 |
| Therapeutic anticoagulant, yes, n (%) | 18 (3.4) | 11 (2.4) | 7 (1.8) | 12 (3.0) | 0.482 |
| Mechanical prophylaxis, yes, n (%) | 530 (100.0) | 465 (100.0) | 387 (100.0) | 405 (100.0) | - |
| Chemoprophylaxis type, LMWH, n (%) | 446 (84.2) | 379 (81.5) | 292 (75.5) | 328 (81.0) | 0.044 |

IQR: Interquartile range, LMWH: Low molecular weight heparin, SD: Standard deviation.

*P* value corrected for multiple comparison using Bonferroni correction.

**Table S4.** Baseline characteristics for major ventral hernia surgery cohort

| **Characteristics** | **Time from skin closure to postoperative chemoprophylaxis** | | | | ***p***  **value** |
| --- | --- | --- | --- | --- | --- |
|  | **Quartile 1**  **N=389** | **Quartile 2**  **N=384** | **Quartile 3**  **N=449** | **Quartile 4**  **N=479** |  |
| **Demographics** |  |  |  |  |  |
| Age, mean (SD) | 58.3 (14.4) | 58.0 (13.8) | 60.0 (14.3) | 60.9 (14.0) | 0.689 |
| Gender, male, n (%) | 199 (51.2) | 180 (46.9) | 217 (48.3) | 232 (48.4) | 0.685 |
| Body mass index, kg/m^2^, mean (SD) | 32.6 (6.9) | 32.5 (6.9) | 32.1 (6.5) | 31.9 (6.3) | 0.384 |
| Caprini score, median (IQR) | 4 (4-5) | 4 (4-5) | 5 (4-5) | 5 (4-5) | 0.109 |
| **Operative details** |  |  |  |  |  |
| Anesthesia type, n (%) |  |  |  |  | 0.832 |
| General only | 371 (95.4) | 366 (95.3) | 434 (96.7) | 460 (96.0) |  |
| Regional only | 2 (0.5) | 3 (0.8) | 1 (0.2) | 4 (0.8) |  |
| General and regional | 16 (4.1) | 15 (3.9) | 14 (3.1) | 15 (3.1) |  |
| Operative approach, n (%) |  |  |  |  | 0.328 |
| Open | 343 (88.2) | 318 (82.8) | 365 (81.3) | 395 (82.5) |  |
| Laparoscopic | 46 (11.8) | 66 (17.2) | 84 (18.7) | 84 (17.5) |  |
| Surgeon seniority, n (%) |  |  |  |  | 0.979 |
| Consultant | 239 (61.4) | 238 (62.0) | 274 (61.0) | 290 (60.5) |  |
| Fellow | 34 (8.7) | 28 (7.3) | 41 (9.1) | 48 (10.0) |  |
| Registrar | 109 (28.0) | 108 (28.1) | 124 (27.6) | 131 (27.3) |  |
| Not documented | 7 (1.8) | 10 (2.6) | 10 (2.2) | 10 (2.1) |  |
| Hernia type, n (%) |  |  |  |  | 0.008 |
| Incisional | 246 (63.2) | 300 (78.1) | 341 (75.9) | 391 (81.6) |  |
| Primary | 143 (36.8) | 84 (21.9) | 108 (24.1) | 88 (18.4) |  |
| Hernia location, n (%) |  |  |  |  | 0.040 |
| Midline | 286 (73.5) | 247 (64.3) | 303 (67.5) | 354 (73.9) |  |
| Off midline | 103 (26.5) | 137 (35.7) | 146 (32.5) | 125 (26.1) |  |
| Number of defects, >1, n (%) | 94 (24.2) | 109 (28.4) | 146 (32.5) | 155 (32.4) | 0.100 |
| Bowel in hernia, yes, n (%) | 76 (19.5) | 88 (22.9) | 102 (22.7) | 106 (22.1) | 0.639 |
| First repair, yes, n (%) | 326 (83.8) | 296 (77.1) | 366 (81.5) | 372 (77.7) | 0.400 |
| Fascia closed, yes, n (%) | 269 (69.2) | 292 (76.0) | 330 (73.5) | 366 (76.4) | 0.070 |
| Component separation, yes, n (%) | 11 (2.8) | 19 (4.9) | 15 (3.3) | 23 (4.8) | 0.308 |
| Drain use, yes, n (%) | 97 (24.9) | 131 (34.1) | 158 (35.2) | 163 (34.0) | 0.921 |
| Mesh use, yes, n (%) | 348 (89.5) | 341 (88.8) | 412 (91.8) | 426 (88.9) | 0.435 |
| Mesh type, composite, n (%) | 239 (68.7) | 212 (62.2) | 238 (57.8) | 253 (59.4) | 0.052 |
| Mesh location, n (%) |  |  |  |  | 0.086 |
| On-lay | 70 (20.1) | 77 (22.6) | 96 (23.3) | 115 (27.0) |  |
| In-lay | 14 (4.0) | 11 (3.2) | 17 (4.1) | 16 (3.8) |  |
| Retro-rectus/Sub-lay | 64 (18.4) | 69 (20.2) | 68 (16.5) | 83 (19.5) |  |
| Preperitoneal | 130 (37.4) | 97 (28.4) | 121 (29.4) | 110 (25.8) |  |
| Intraperitoneal | 70 (20.1) | 87 (25.5) | 110 (26.7) | 99 (23.2) |  |
| Mesh anchorage, n (%) |  |  |  |  | 0.097 |
| Sutures only | 281 (80.7) | 260 (76.2) | 306 (74.3) | 306 (71.8) |  |
| Tacks only | 32 (9.2) | 39 (11.4) | 38 (9.2) | 54 (12.7) |  |
| Glue only | 0 (0.0) | 0 (0.0) | 2 (0.5) | 2 (0.5) |  |
| Combination | 35 (10.1) | 42 (12.3) | 66 (16.0) | 64 (15.0) |  |
| **Perioperative details** |  |  |  |  |  |
| ASA score, median (IQR) | 2 (2-3) | 2 (2-3) | 2 (2-3) | 2 (2-3) | 0.101 |
| Surgical duration, min, mean (SD) | 106.1 (70.2) | 113.7 (57.6) | 110.4 (58.9) | 117.5 (66.4) | 0.055 |
| Length-of-stay, days, mean (SD) | 2.5 (3.6) | 3.2 (3.8) | 3.1 (4.1) | 3.9 (4.8) | 0.067 |
| Antiplatelet agent use, yes, n (%) | 2.5 (3.6) | 3.2 (3.8) | 3.1 (4.1) | 3.9 (4.8) | 0.192 |
| Therapeutic anticoagulant use, yes, n (%) | 11 (2.8) | 19 (4.9) | 25 (5.6) | 37 (7.7) | 0.064 |
| Mechanical prophylaxis, yes, n (%) | 364 (93.6) | 353 (91.9) | 410 (91.3) | 449 (93.7) | 0.425 |
| Chemoprophylaxis type, LMWH, n (%) | 337 (86.6) | 333 (86.7) | 394 (87.8) | 425 (88.7) | 0.761 |

IQR: Interquartile range, LMWH: Low molecular weight heparin, SD: Standard deviation.

*P* value corrected for multiple comparison using Bonferroni correction.

**Table S5.** Baseline characteristics for major abdominal visceral resection cohort

| **Characteristics** | **Time from skin closure to postoperative chemoprophylaxis** | | | | ***P***  **value** |
| --- | --- | --- | --- | --- | --- |
|  | **Quartile 1**  **N=163** | **Quartile 2**  **N=171** | **Quartile 3**  **N=140** | **Quartile 4**  **N=194** |  |
| **Demographics** |  |  |  |  |  |
| Age, mean (SD) | 63.8 (12.0) | 65.9 (10.6) | 64.6 (11.1) | 62.2 (14.0) | 0.136 |
| Gender, male, n (%) | 74 (45.4) | 76 (44.4) | 76 (54.3) | 106 (54.6) | 0.104 |
| Body mass index, kg/m2, mean (SD) | 28.8 (6.4) | 27.9 (6.0) | 28.6 (6.7) | 27.8 (5.7) | 0.320 |
| Caprini score, median (IQR) | 6 (5-7) | 6 (5-7) | 6 (5-7) | 6.5 (5-7) | 0.543 |
| **Operative details** |  |  |  |  |  |
| Anesthesia type, n (%) |  |  |  |  | 0.160 |
| General only | 119 (73.0) | 148 (86.5) | 114 (81.4) | 153 (78.9) |  |
| General and regional | 44 (27.0) | 23 (13.5) | 26 (18.6) | 41 (21.1) |  |
| Operation type, n (%) |  |  |  |  | 0.074 |
| Oesophagectomy | 7 (4.3) | 2 (1.2) | 4 (2.9) | 11 (5.7) |  |
| Gastrectomy | 4 (2.5) | 3 (1.8) | 3 (2.1) | 4 (2.1) |  |
| Splenectomy | 1 (0.6) | 2 (1.2) | 1 (0.7) | 3 (1.5) |  |
| Liver resection | 23 (14.1) | 13 (7.6) | 15 (10.7) | 26 (13.4) |  |
| Bile duct resection | 2 (1.2) | 0 (0.0) | 1 (0.7) | 5 (2.6) |  |
| Pancreatectomy | 29 (17.8) | 11 (6.4) | 8 (5.7) | 12 (6.2) |  |
| Small bowel resection | 1 (0.6) | 5 (2.9) | 7 (5.0) | 6 (3.1) |  |
| Colon resection | 29 (17.8) | 61 (35.7) | 55 (39.3) | 56 (28.9) |  |
| Rectal resection | 64 (39.3) | 72 (42.1) | 42 (30.0) | 61 (31.4) |  |
| Hartmann reversals | 3 (1.8) | 2 (1.2) | 4 (2.9) | 10 (5.2) |  |
| Operative approach, n (%) |  |  |  |  | 0.072 |
| Open | 70 (42.9) | 44 (25.7) | 53 (37.9) | 66 (34.0) |  |
| Laparoscopic | 93 (57.1) | 127 (74.3) | 87 (62.1) | 128 (66.0) |  |
| Surgeon seniority, n (%) |  |  |  |  | 0.024 |
| Consultant | 99 (60.7) | 86 (50.3) | 81 (57.9) | 137 (70.6) |  |
| Fellow | 58 (35.6) | 76 (44.4) | 47 (33.6) | 49 (25.3) |  |
| Registrar | 6 (3.7) | 9 (5.3) | 12 (8.6) | 8 (4.1) |  |
| **Perioperative details** |  |  |  |  |  |
| Malignant pathology, yes, n (%) | 125 (76.7) | 124 (72.5) | 86 (61.4) | 127 (65.5) | 0.128 |
| ASA score, median (IQR) | 3 (2-3) | 3 (2-3) | 3 (2-3) | 3 (2-3) | 0.798 |
| Surgical duration, min, mean (SD) | 309.4 (133.1) | 222.1 (78.6) | 250.6 (139.8) | 256.3 (122.5) | 0.052 |
| Length-of-stay, days, mean (SD) | 13.2 (30.6) | 8.4 (8.7) | 12.5 (22.7) | 12.6 (25.0) | 0.229 |
| Antiplatelet agent use, yes, n (%) | 25 (15.3) | 22 (12.9) | 27 (19.3) | 26 (13.4) | 0.386 |
| Therapeutic anticoagulant use, yes, n (%) | 12 (7.4) | 12 (7.0) | 6 (4.3) | 14 (7.2) | 0.673 |
| Mechanical prophylaxis, yes, n (%) | 163 (100.0) | 169 (98.8) | 140 (100.0) | 194 (100.0) | 0.120 |
| Chemoprophylaxis type, LMWH, n (%) | 150 (92.0) | 166 (97.1) | 132 (94.3) | 192 (99.0) | 0.056 |

IQR: Interquartile range, LMWH: Low molecular weight heparin, SD: Standard deviation.

*P* value corrected for multiple comparison using Bonferroni correction.

**Table S6.** Baseline characteristics for cholecystectomy cohort

| **Characteristics** | **Time from skin closure to postoperative chemoprophylaxis** | | | | ***P***  **value** |
| --- | --- | --- | --- | --- | --- |
|  | **Quartile 1**  **N=111** | **Quartile 2**  **N=153** | **Quartile 3**  **N=210** | **Quartile 4**  **N=99** |  |
| **Demographics** |  |  |  |  |  |
| Age, mean (SD) | 51.1 (15.7) | 52.0 (16.9) | 51.5 (17.3) | 56.7 (16.3) | 0.517 |
| Gender, male, n (%) | 40 (36.0) | 55 (35.9) | 71 (33.3) | 32 (32.3) | 0.917 |
| Body mass index, kg/m^2^, mean (SD) | 29.0 (6.9) | 29.2 (6.4) | 29.9 (8.2) | 31.4 (17.9) | 0.331 |
| Caprini score, median (IQR) | 4 (3-5) | 4 (3-5) | 4 (3-5) | 4 (3-5) | 0.157 |
| **Operative details** |  |  |  |  |  |
| Anesthesia type, n (%) |  |  |  |  | - |
| General only | 111 (100.0) | 153 (100.0) | 210 (100.0) | 99 (100.0) |  |
| General and regional | 0 (0.0) | 0 (0.0) | 0 (0.0) | 0 (0.0) |  |
| Operative approach, n (%) |  |  |  |  | 0.104 |
| Open | 4 (3.6) | 0 (0.0) | 2 (1.0) | 5 (5.1) |  |
| Laparoscopic | 107 (96.4) | 153 (100.0) | 208 (99.0) | 94 (94.9) |  |
| Surgeon seniority, n (%) |  |  |  |  | 0.068 |
| Consultant | 37 (33.3) | 49 (32.0) | 73 (34.8) | 48 (48.5) |  |
| Fellow | 23 (20.7) | 23 (15.0) | 29 (13.8) | 15 (15.2) |  |
| Registrar | 51 (45.9) | 81 (52.9) | 108 (51.4) | 36 (36.4) |  |
| **Perioperative details** |  |  |  |  |  |
| Benign pathology, yes, n (%) | 111 (100.0) | 152 (99.3) | 209 (99.5) | 96 (97.0) | 0.080 |
| ASA score, median (IQR) | 2 (1-2) | 2 (1-2) | 2 (1-2) | 2 (2-3) | 0.055 |
| Surgical duration, min, mean (SD) | 87.7 (38.1) | 92.6 (59.1) | 84.8 (35.3) | 90.5 (41.1) | 0.392 |
| Length-of-stay, days, mean (SD) | 1.5 (1.3) | 1.6 (1.7) | 1.5 (1.8) | 2.4 (2.9) | 0.050 |
| Antiplatelet agent use, yes, n (%) | 10 (9.0) | 13 (8.5) | 17 (8.1) | 10 (10.1) | 0.948 |
| Therapeutic anticoagulant use, yes, n (%) | 4 (3.6) | 6 (3.9) | 8 (3.8) | 7 (7.1) | 0.548 |
| Mechanical prophylaxis, yes, n (%) | 110 (99.1) | 151 (98.7) | 203 (96.7) | 93 (93.9) | 0.078 |
| Chemoprophylaxis type, LMWH, n (%) | 105 (94.6) | 146 (95.4) | 197 (93.8) | 96 (97.0) | 0.680 |

IQR: Interquartile range, LMWH: Low molecular weight heparin, SD: Standard deviation.

*P* value corrected for multiple comparison using Bonferroni correction.
